# Celecoxib effectively inhibits the formation of joint adhesions

**DOI:** 10.3892/etm.2013.1336

**Published:** 2013-10-09

**Authors:** FENGFENG LI, BIN HE, SHEN LIU, CUNYI FAN

**Affiliations:** 1 Department of Orthopedics, Sixth Affiliated People’s Hospital of Shanghai Jiaotong University, Shanghai 200233, P.R. China; 2 Department of Orthopedics, Second Affiliated Hospital of Nanjing Medical College, Nanjing 210011, P.R. China

**Keywords:** celecoxib, ibuprofen, nonselective cyclooxygenase-2 enzyme inhibitor, selective cyclooxygenase-2 enzyme inhibitor, joint adhesion

## Abstract

The present study investigated the effectiveness of celecoxib in preventing the formation of joint adhesions. Rabbit models of joint adhesion were created and the rabbits in two treatment groups were orally administered celecoxib or ibuprofen (as a positive control) for 30 days. Rabbits in the control group did not receive any treatment. Following the 30-day experimental period, the inhibitory effects of celecoxib and ibuprofen on the formation of joint adhesion were assessed using a number of methods, including the study of macroscopic appearance, histology and contracture angle. Thick fibrous adhesions developed in the knees of the rabbits in the control group. By contrast, few adhesions were observed in the two treatment groups, and those observed were soft, weak and easily stretched. Fewer adhesions were observed in the rabbits treated with celecoxib than in the rabbits in the other groups. The adhesion scores and contracture angles in the celecoxib (P<0.001) and ibuprofen (0.001<P<0.0025) groups were significantly lower than those of the control group. Moreover, the adhesion scores and contracture angles were significantly lower in the celecoxib group than in the ibuprofen group (0.025<P<0.05). Histologically, the adhesion tissues in the two treatment groups, particularly in the celecoxib group, were loose and thin with sparse fiber formation. The cell densities in the two treatment groups, of which the ibuprofen group had higher cell densities (0.025<P<0.05), were significantly lower than those in the control group (celecoxib group, P<0.001; ibuprofen group, 0.001<P<0.0025). These results indicated that celecoxib effectively inhibited the formation of joint adhesions and therefore may provide a novel and potent approach for their prevention.

## Introduction

The formation of fibrous joint adhesions following surgery or trauma severely restricts functional recovery and is a major problem in the field of orthopedics. The efficacy of certain pharmaceutical agents in the prevention of adhesion formation, following local administration, has been examined (1–4). These attempts have achieved limited success and thus adhesion formation remains an unsolved problem. A number of selective and nonselective cyclooxygenase (COX)-2 enzyme inhibitors have been shown to reduce adhesion formation via inhibition of inflammatory responses involving polymorphonuclear leukocytes, macrophages, fibrin, fibroblasts and new blood vessel formation. However, as a result of their limited effects, COX-2 inhibitors have rarely been applied clinically (5–11).

Celecoxib, a selective COX-2 inhibitor, has previously been shown to produce a maximal reduction in intra-abdominal adhesion formation compared with ibuprofen and other nonselective COX-2 inhibitors (12,13). In addition to its COX-2 inhibitory effects, and unlike other COX enzyme inhibitors, celecoxib also possesses antiangiogenic and antifibroblastic properties (7,14). These additional properties may contribute to the greater efficiency of celecoxib in reducing adhesion formation compared with nonselective COX-2 enzyme inhibitors. Therefore, celecoxib may provide a promising therapy for joint adhesions, as all types of adhesions are considered to arise through the same mechanism. The aim of the present study was to demonstrate marked inhibition of joint adhesion formation by oral administration of celecoxib. A rabbit model that accurately mimicked the features of human joint adhesion was created and the inhibitory effects of celecoxib on the formation of adhesions were assessed in this model. The effects were investigated from a number of aspects, including joint flexion contracture angle, macroscopic appearance, histology and collagen content. To provide a positive comparison, ibuprofen was also administered in this study due to its maximal antiadhesion effect among the nonselective COX-2 inhibitors (13,15).

## Materials and methods

### Animal model

**Figure 1:**
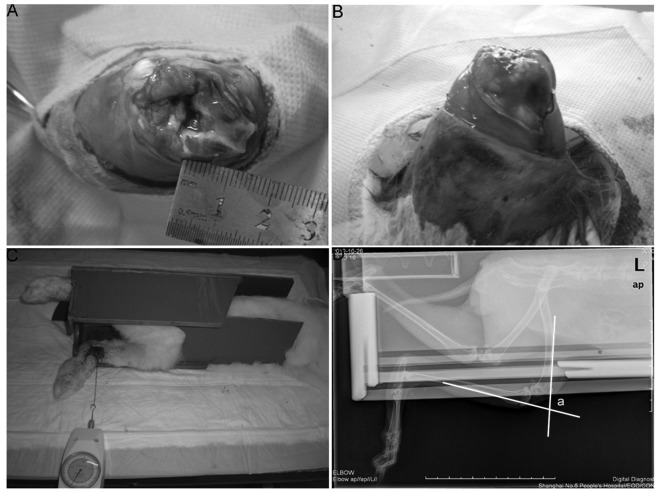
Creation of the animal model and measurement of the flexion contracture angle. (A) Removal of an osteochondral portion of the condyle. (B) Immobilization of the knee joint with a Kirschner wire. (C) Extension of the knee joint by pulling the suture with a tension of 4.9 N while the left thigh was firmly fixed to the table. The tension was produced using a dynamometer. (D) Radiography was performed to determine the contracture angle. A 1–0 suture was tied to the left ankle and the direction of the suture was maintained perpendicular to the tibial axis. a, flexion contracture angle.

A total of 60 female New Zealand White rabbits (weight, 2.5–3.0 kg; age, 3–4 months) were purchased from the Shanghai Laboratory Animal Center (Chinese Academy of Sciences, Shanghai, China). All experimental procedures were approved by the Research Ethics Committees of the Sixth Affiliated People’s Hospital of Shanghai Jiaotong University (Shanghai, China). The rabbits were randomly assigned to one of the following three groups (n=20 per group): Celecoxib, ibuprofen and control groups. Using intravenous pentobarbital sodium (Dainihon, Osaka, Japan) to achieve anesthesia, the left knee joint was prepared for surgery under aseptic conditions. Following a skin incision, the knee was opened using a lateral parapatellar approach, and the medial and lateral sides of the femoral condyle were exposed. A partial capsulotomy and synovectomy were performed using an osteotome and an osteochondral portion of the condyle (~5×10 mm) was removed to ensure the underlying cancellous bone was exposed (Fig. 1A). Each limb that was subjected to surgery underwent knee joint immobilization at 140° of flexion with a Kirschner wire (Zimmer, Shanghai, China) for 30 days (Fig. 1B). Rabbits were housed in individual cages in which motion was not limited. They had access to food and water *ad libitum*.

### Drug treatment

Following recovery from anesthesia, all rabbits were administered prophylactic cefazolin and buprenorphine (Shanghai Pharma, Shanghai, China) for pain management as required. In addition, the rabbits in the two treatment groups received oral celecoxib (2.86 mg/kg twice a day; Pfizer, Shanghai, China) or ibuprofen (3.81 mg/kg three times a day; GSK, Shanghai, China), commencing on the day of surgery, for 30 days. The rabbits in the control group did not receive oral celecoxib or ibuprofen.

### Macroscopic evaluation

Thirty days subsequent to surgery, eight rabbits in each group were sacrificed by intravenous administration of an overdose of pentobarbital sodium (Dainihon) and the Kirschner wires were removed. The knee joint of each rabbit was exposed via a parapatellar skin incision and held at 140° of flexion. The presence and severity of intra-articular adhesions were semiquantitatively assessed by blinded observers using a severity score scale of 0–4 (16) as follows: Grade 0, no adhesions; grade 1, filmy weak adhesions, easily exfoliated by light preparation with forceps; grade 2, mild adhesions, easily exfoliated by moderate preparation with forceps; grade 3, moderately dense adhesions, which may be partly exfoliated by strong preparation with forceps; grade 4, dense fibrous adhesions, which may not be exfoliated via strong preparation with forceps.

### Histological evaluation of adhesion tissues

Following macroscopic evaluation of the adhesions, intra-articular adhesion tissues were harvested from the knee and fixed in 10% neutral buffered formalin for one week. Subsequently, the tissues were embedded in paraffin and 6-μm sections were prepared. The sections were mounted on silane-coated slides and stained with hematoxylin and eosin (H&E). The sections were then observed under a microscope (VanoxAH-2; Olympus, Tokyo, Japan), and fibrosis, predominant cells and cell densities were evaluated. Image-Pro Plus (Media Cybernetics, Silver Spring, MD, USA) was used to analyze cell numbers.

### Measurement of the flexion contracture angle

Following macroscopic and histological evaluations, the animals remaining in each group were also sacrificed via administration of an overdose of pentobarbital sodium (Dainihon), and were immediately subjected to biomechanical evaluations. Following removal of the Kirschner wire, a thick silk thread was hooked onto the lower left leg, 10 cm distal from the knee, and an extension of 5.5 N was applied perpendicular to the tibia by pulling the thread with a dynamometer (Fig. 1C). The animal was laid on the X-ray table on its left side and the flexion contracture angle was determined on a lateral view radiograph of the left knee (Fig. 1D, angle ‘a’). All measurements were made within 15 min of sacrifice.

### Statistical analysis

Statistical analyses of the differences among the groups were performed using one-way analysis of variance (ANOVA) and a post-hoc Student-Newman-Keuls test. The data are presented as the mean ± standard deviation (SD). P<0.05 was considered to indicate a statistically significant difference. All statistical analyses were conducted using SPSS 11.0 (SPSS, Inc., Chicago, IL, USA).

## Results

In general, the rabbits adapted to Kirschner wire immobilization well during the 30 days of the experimental period. However, of the 60 rabbits used in this study, one animal in the celecoxib group died on day 7 of unknown causes and two animals suffered from wound infection (one each from the ibuprofen and control groups). In addition, the fixation of two animals became loose in the celecoxib and ibuprofen groups, respectively. These seven animals mentioned were excluded from the study. All other animals gained weight and appeared healthy, with no signs of impaired wound healing.

**Figure 2:**
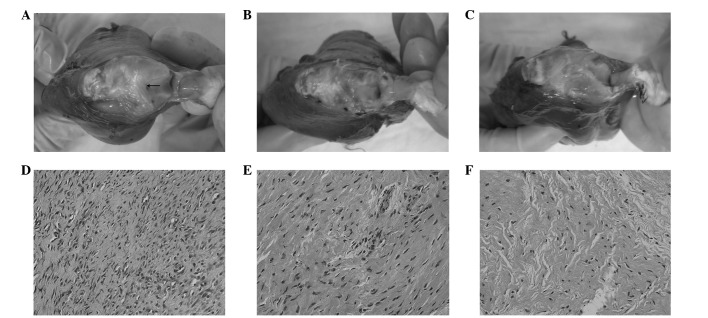
Photographs and histological analyses of adhesions in knee joints. Compared with the adhesions in (A and D) the control group and (B and E) the ibuprofen group, the adhesions in (C and F) the celecoxib group were reduced in number and volume. Sections were stained with hematoxylin and eosin (original magnification, ×100).

At 30 days following surgery, thick fibrous adhesions had developed in the knees of the rabbits in the control group (Fig. 2A). By contrast, the adhesions in the two treatment groups were few, soft, weak and easily stretched (Fig. 2B and C). Furthermore, fewer adhesions were observed in the rabbits treated with celecoxib compared with those treated with ibuprofen (Fig. 2C). Accordingly, the adhesion scores in the ibuprofen (0.001<P<0.0025) and celecoxib (P<0.001) groups were significantly lower than those in the control group (Table I). Moreover, the adhesion scores were significantly lower in the celecoxib group than those in the ibuprofen group (Table I; 0.025<P<0.05).

Histologically, the adhesion tissues in the control group were dense, thick and fibrous (Fig. 2D), while those in the ibuprofen group and particularly in the celecoxib group were loose and thin with sparse fiber formation (Fig. 2E and F). In all three groups, the predominant cells were considered to be fibroblasts and few inflammatory cells were observed. The cell densities in the celecoxib (range, 117–130; mean, 124; P<0.001) and the ibuprofen (range, 151–165; mean, 158; 0.001<P<0.0025) groups were significantly lower than those in the control group (range, 220–239; mean, 229; Table II). Moreover, the cell densities were lower in the celecoxib group than those in the ibuprofen group (Table II; 0.025<P<0.05).

As exhibited in Table III, the flexion contracture angles in the control group ranged between 112 and 125° (mean, 120°). Despite the differences between the two treatment groups, the contracture angles in the celecoxib group (range, 21–32°; mean, 26.4°; P<0.001) and the ibuprofen group (range, 40–48°; mean, 44.2°; 0.001<P<0.0025) were significantly lower than those in the control group. Moreover, the contracture angles were significantly lower in the celecoxib group than in the ibuprofen group (0.025<P<0.05).

## Discussion

Adhesion development is a process that involves tissue fibrosis, with inflammatory responses involving polymorphonuclear leukocytes, macrophages, fibrin, fibroblasts and new blood vessel formation. The fibroblasts in adhesions express COX-2 enzymes, while those in non-adhesion-bearing areas do not (17). Consequently, certain selective and nonselective COX-2 enzyme inhibitors may, to an extent, inhibit adhesion formation. In addition, the formation of all types of adhesions is partly dependent on angiogenesis (18,19), and there is experimental evidence indicating that COX-2-induced prostaglandins may modulate fibroblast growth factor- and vascular endothelial growth factor-induced angiogenesis (20). Thus, the COX-2 inhibitor, celecoxib, may selectively inhibit the angiogenesis associated with newly forming adhesions via a COX-2-based mechanism (8), thereby producing a reduction in intra-abdominal adhesion formation (13).

In view that the same mechanism underlies the formation of different adhesion types, celecoxib may represent a promising therapy for joint adhesions. In the present study, the inhibitory effects of celecoxib on the formation of joint adhesions were demonstrated *in vivo* from different aspects, including adhesion score, histology, collagen content and joint contracture angle. The results of the histological and biochemical analyses of the adhesion tissues were closely correlated with those of the macroscopic and biomechanical evaluations. For example, collagen content is one of the key factors determining the mechanical strength of repair tissues (21). The collagen type ratio is another determinant of tissue strength, and a higher proportion of type III collagen is assumed to decrease the strength by reducing the fibril diameter, which is closely correlated with the strength of connective tissues (22,23). Thus, the histological and biochemical alterations in the adhesion tissues may serve to improve joint contracture.

In the present study, a clinically applicable dosing regimen of the tested drugs was used. Furthermore, celecoxib was administered via orogastric feeding on a daily basis for 30 days, thereby effectively simulating a clinical setting. Using an oral daily dosing schedule, it was demonstrated that ibuprofen and celecoxib reduced the formation of joint adhesions. However, the inhibitory effect of ibuprofen on adhesion formation was not as extensive as that of celecoxib. Furthermore, the nonselective COX-2 inhibitor, ibuprofen, has the disadvantage of causing harmful side effects associated with COX-1 inhibition, including gastrointestinal and renal complications, as well as bleeding, as a result of inhibition of platelet aggregation. Furthermore, it appears that long-term administration and high doses of COX-2 inhibitors increase the risk of adverse cardiovascular events (24–26). However, patients at risk of joint adhesion formation may represent a new cohort suitable for celecoxib treatment due to the potentially low risk-to-benefit ratio. Firstly, it appeared that long-term celecoxib administration was not required to prevent adhesion formation, since joint adhesions are formed within 30 days, and a 30-day treatment led to long-term adhesion prevention in the present study. Secondly, routine doses of celecoxib are likely to be required for efficacy in humans, thereby minimizing the increased risk of cardiovascular events associated with the high doses used for treating diseases such as rheumatoid arthritis. Furthermore, celecoxib has a lower toxicity profile than other COX-2 inhibitors (27).

An animal model that accurately mimics the features of human joint adhesions is essential for the preclinical evaluation of the efficacy and safety of this form of drug therapy. An intra-articular adhesion model has been developed in rabbits and is currently being used to evaluate several methods for inhibiting the formation of joint adhesions (1,28). In the present study, a similar type of intra-articular adhesion model in rabbits was used to evaluate the effects of celecoxib for the treatment of joint adhesions. The high incidence rate (100%) of joint adhesion formation in the control group indicated that the experimental surgical procedure used in the present study was appropriate for this type of study. In addition, the adhesions developed in the articular cavity of this model; therefore, the adhesion tissues were of sufficient volume to enable biochemical analyses without contamination by other tissues.

The present study demonstrated that oral delivery of celecoxib ameliorated joint adhesion formation effectively and safely in a rabbit model. The study provides a novel and promising strategy that may serve as an alternative treatment for the prevention of joint adhesion formation. Further studies involving larger animals are required to support this hypothesis. In addition, clinical trials are required to confirm the beneficial properties of celecoxib in reducing joint adhesions.

## Figures and Tables

**Table I:** Adhesion scores of the rabbits in the control and treatment groups.

Group	Number	Adhesion scores	P-value^a^	P-value^b^
Control	8	3.6±0.4	NA	NA
Ibuprofen	8	1.9±0.3	0.001<P<0.0025	NA
Celecoxib	8	1.4±0.3	P<0.001	0.025<P<0.05

a Compared with the control group;

b Compared with the ibuprofen group.

All animals in each group were used for this evaluation. Data are presented as the mean ± standard deviation. Values of P<0.05 are considered to indicate a statistically significant difference. NA, not applicable.

**Table II:** Cell densities of the rabbits in the control and treatment groups.

Group	Number	Total cells (per hpf)	P-value^a^	P-value^b^
Control	8	229±19	NA	NA
Ibuprofen	8	158±13	0.001<P<0.0025	NA
Celecoxib	8	124±9	P<0.001	0.025<P<0.05

a Compared with the control group;

b Compared with the ibuprofen group.

All animals in each group were used for this evaluation. Data are presented as the mean ± standard deviation. Values of P<0.05 are considered to indicate a statistically significant difference. hpf, high-power field; NA, not applicable.

**Table III:** Contracture angles of the rabbits in the control and treatment groups.

Group	Number	Contracture angles (°)	P-value^a^	P-value^b^
Control	11	120.0±11.2	NA	NA
Ibuprofen	9	44.2±4.4	0.001<P<0.0025	NA
Celecoxib	9	26.4±3.4	P<0.001	0.025<P<0.05

a Compared with the control group;

b Compared with the ibuprofen group.

All animals in each group were used for this evaluation. Data are presented as the mean ± standard deviation. Values of P<0.05 are considered to indicate a statistically significant difference. NA, not applicable.
